# Spectral Super-Resolution for High Dynamic Range Images

**DOI:** 10.3390/jimaging9040083

**Published:** 2023-04-14

**Authors:** Yuki Mikamoto, Yoshiki Kaminaka, Toru Higaki, Bisser Raytchev, Kazufumi Kaneda

**Affiliations:** Graduate School of Advance Science and Engineering, Hiroshima University, Higashi-Hiroshima 739-8527, Japan

**Keywords:** Spectral Super-Resolution, deep learning, image-based lighting, spectral rendering, High Dynamic Range image

## Abstract

The images we commonly use are RGB images that contain three pieces of information: red, green, and blue. On the other hand, hyperspectral (HS) images retain wavelength information. HS images are utilized in various fields due to their rich information content, but acquiring them requires specialized and expensive equipment that is not easily accessible to everyone. Recently, Spectral Super-Resolution (SSR), which generates spectral images from RGB images, has been studied. Conventional SSR methods target Low Dynamic Range (LDR) images. However, some practical applications require High Dynamic Range (HDR) images. In this paper, an SSR method for HDR is proposed. As a practical example, we use the HDR-HS images generated by the proposed method as environment maps and perform spectral image-based lighting. The rendering results by our method are more realistic than conventional renderers and LDR SSR methods, and this is the first attempt to utilize SSR for spectral rendering.

## 1. Introduction

The images we commonly use are RGB images that contain three pieces of information: red, green, and blue. On the other hand, hyperspectral (HS) images retain wavelength information. HS images are utilized in various fields due to their rich information content, but acquiring them requires the specialized and expensive equipment that is not easily accessible to everyone.

Recently, Spectral Super-Resolution (SSR), which generates HS images from RGB images, has been studied in computer vision. These studies mainly employ deep learning to generate HS images from RGB images, which potentially makes HS images more accessible. However, conventional SSR methods are designed for Low Dynamic Range (LDR) images, while some applications require High Dynamic Range (HDR) images.

For example, Image-Based Lighting (IBL) [1] requires HDR circumference images as environment maps. In rendering, spectral data can generate more physically correct images than RGB data, but preparing real HDR-HS environment maps is troublesome because it needs a long capturing time and complicated processing. SSR methods seem suitable for the rendering, but conventional SSR methods that target LDR produce lower-quality results due to dynamic range limitations.

In this paper, we propose an SSR method for HDR-HS images. In order to realize SSR compatibility with HDR, there are two problems to be solved: It is very difficult to prepare training data and the dynamic range of the luminance in HDR images is quite extensive, and the upper limit cannot be specified. To solve both problems, the HS image is divided into luminance and spectral similarity, and the network only learns the spectral similarity of the HS image. Then, the luminance is restored in the HDR-RGB image to generate the HDR-HS image. To further solve the latter problem, we apply the tone mapping function, which is typically used to display HDR images, to an HDR image as preprocessing to normalize luminance.

Since there are no datasets for SSR supporting HDR, we create a pseudo-HDR-RGB image from an existing HS dataset for training the network. In addition, we create real HDR-HS images captured by using a hyperspectral camera to evaluate the practicality of the network trained by pseudo-HDR images. Furthermore, we extend a PBRT renderer [2] to perform spectral IBL with HDR-HS images generated by the proposed method.

## 2. Related Work

### 2.1. Spectral Super-Resolution

The spectral upsampling method for rendering converts RGB values into a spectrum, while the SSR method converts RGB images into HS images. Hence, SSR methods enable spectral upsampling that considers color and objects.

The early SSR method using only RGB images is dictionary learning using the K-SVD algorithm [3] by Arad et al. [4]. This research shows that the performance of the SSR method is comparable to the method of up-converting the resolution of a low spatial resolution spectral image using a high spatial resolution RGB image. After the research, SSR methods using only RGB images have become mainstream.

Galliani et al. [5] focused on the superior performance of CNN in spatial super-resolution [6] and colorization of grayscale images [7,8]; they also used the fully convolutional DenseNets [9] for SSR, originally designed for semantic segmentation and it is the first method using CNN for SSR and outperforms conventional methods using dictionary learning. Since then, various kinds of CNN-based methods have been proposed.

In order to further promote SSR, the “NTIRE 2018 Challenge on Spectral Reconstruction from RGB Images” [10] was held as the first competition. The HSCNN-D proposed by Shi et al. [11], which uses dense structure and path-widening fusion, won the “Clean” track in this competition. Due to the success of the first challenge, the second challenge was held in 2020 [12]. In the second competition, the Adaptive Weighted Attention Network (AWAN) proposed by Li et al. [13] won in the “Clean” track. This method uses spatial contextual information and correlations between channels.

Most SSR methods, including those mentioned above, are end-to-end learning, where the networks learn spectral values directly rather than spectral similarity. On the other hand, Sakamoto et al. [14] proposed a novel method based on decomposing luminance and chrominance, where spectral values are decomposed into luminance components, and the similarity of spectral distribution, and the network learns only the spectral similarity. The decomposition method drastically improves the accuracy compared to the end-to-end learning method because the network concentrates on learning only the distribution shape of the spectrum. Since the spectrum output from the network does not have the original luminance component, luminance restoration is performed on it as a post-process.

Mikamoto et al. [15] realized SSR with a basis function representation of spectral distribution. The network outputs a set of basis function coefficients representing the spectral distribution in this method. This study revealed which basis functions work well depending on the spectral distributions. Based on this study, Mikamoto et al. [16] further improved the accuracy by using a multiple-branch network with multiple basis functions.

### 2.2. Image-Based Lighting

For realistic image synthesis, IBL has been proposed by Debevec [1]. IBL uses HDR images taken in the real world as environment maps that record light source information from the surroundings. RGB images are usually used for environment maps of IBL, but RGB rendering cannot accurately represent highly wavelength-dependent phenomena such as thin-film interference phenomena. In order to perform spectral rendering, it is necessary to capture HS environment maps or convert an RGB image into an HS image.

Some methods of capturing HS environment maps have been proposed. Hirai et al. [17] realized an HDR omnidirectional spectral imaging system that utilized two programmable high-speed cameras with programmable rotating tables and different color filters. This method captured the HS image with a spatial resolution of 7260×3630 in approximately three minutes, but it has the problem that image registration sometimes fails due to the color artifact caused by the occlusion around close-range objects.

Morimoto et al. [18] developed a hyperspectral imaging system consisting of a hyperspectral camera with a tunable liquid-crystal filter, a mirror sphere, and a control PC. Shiwen et al. [19] proposed a portable hyperspectral imaging system using a mirrored chrome sphere and a hyperspectral camera. Both systems are one of the few capable of capturing HS environment maps, but they have problems with low spatial resolution and long capturing time.

Capturing HS environment maps and using them for rendering would be preferred, but this strategy suffers from low spatial resolution, long capturing time, and complicated procedures. Thus, physically based renderers implement spectrum upsampling methods, such as Smits’s method [20] in the PBRT renderer [2], to convert spectra from RGB. However, these methods do not reproduce the actual spectrum of the object since they only consider colors.

## 3. Materials and Methods

### 3.1. Spectral Super-Resolution with High Dynamic Range

IBL requires HDR images, although conventional SSR methods target LDR. We extended the SSR method to be able to handle HDR images. Two problems should be solved to extend the SSR method to HDR.

**Figure 1:**
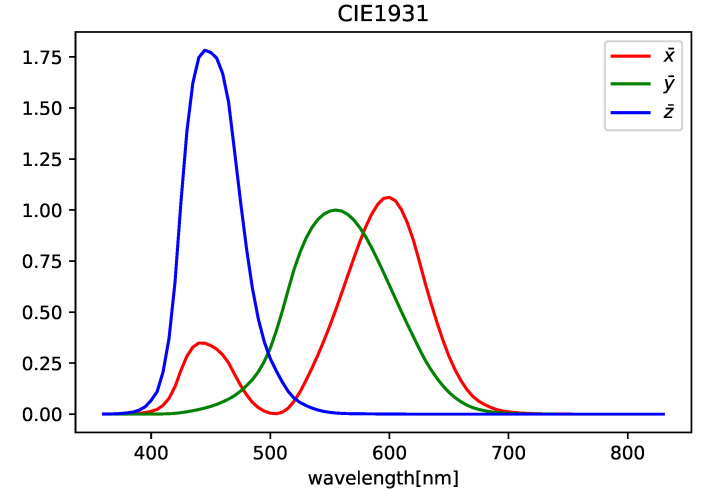
CIE1931 color matching function.

First, there is no dataset suitable for SSR with HDR-HS images. Publicly available datasets for SSR mostly consist of a set of HS images and corresponding LDR-RGB images. For example, the ARAD HS dataset [12] consists of HS images with 12-bit intensity for each wavelength and LDR-RGB images converted using camera response functions. In our research, we created pseudo-HDR-RGB images from spectral images to train the network. The method for creating HDR-RGB images is as follows:

1. Convert the HS images into XYZ images by using the CIE1931 color matching function (see Figure 1);
2. Convert the XYZ images into RGB images by using the transformation matrix;
3. Clip negative values to 0;
4. Store the RGB images in HDR format with a 16-bit floating point (OpenEXR) without normalization and quantization.

We used the following transformation matrix to convert XYZ to sRGB (D65) values:

(1) $$\left[\begin{array}{c} R\\ G\\ B \end{array}\right]=\left[\begin{array}{ccc} 3.2406 & -1.5372 & -0.4986\\ -0.9689 & 1.8758 & 0.0415\\ 0.0557 & -0.2040 & 1.0507 \end{array}\right]\left[\begin{array}{c} X\\ Y\\ Z \end{array}\right].$$

**Figure 2:**
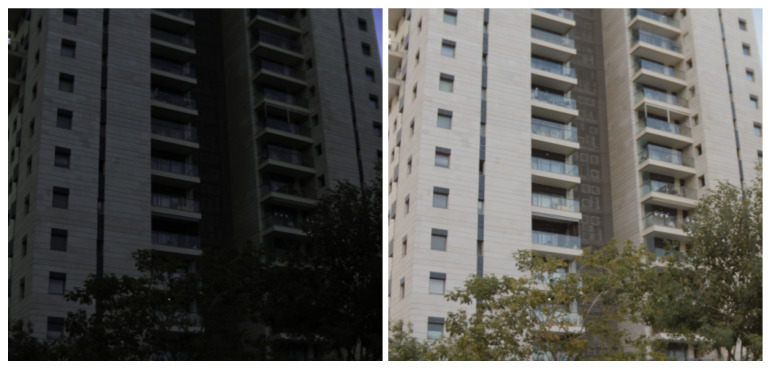
Comparison of the LDR-RGB and the created pseudo-HDR-RGB image. The left image is LDR-RGB, and the right is HDR-RGB.

Figure 2 shows a side-by-side comparison of the LDR-RGB image and the created pseudo-HDR-RGB image in the ARAD HS dataset. From the left figure, the LDR-RGB image cannot be recognized in dark areas. In contrast, it can be seen that these areas are recognized in the pseudo-HDR-RGB image. Thus, we have successfully rescaled the dynamic range of RGB images to match that of HS images. However, one limitation of this method is that the dynamic range of the HS image must be wider than that of the RGB image. If the dynamic range of the HS image is narrower than that of the RGB image, the created RGB image will remain LDR.

Second, the dynamic range of the luminance in HDR images is quite extensive, and the upper limit cannot be specified. In deep learning networks, it is common to normalize input features. The RGB values of LDR images are expressed in 8-bit integers, and it can be considered that they have already normalized. On the other hand, the value of each channel of HDR images preserves the real luminance information, and there is no upper limit to the luminance. In case HDR luminances were directly input to the network, too much attention would be paid to the high luminance areas such as light sources, which would lead to deterioration of accuracy. In fact, some studies that reconstruct HDR from a single LDR image convert the HDR intensity into log space [21,22].

To address the problem, we introduced a simplified version of Reinhard’s tone mapping function [23] to normalize the HDR images. The tone mapping function is expressed by the following equation:

(2) $$f\left(x\right)=\frac{x}{1+x}.$$

### 3.2. Network for Spectral Super-Resolution

The network for SSR is based on Mikamoto et al.’s multiple-branch network [16]. We selected the two-branch network with sigmoid functions and Mexican hat wavelets because the combination of these basis functions gave the best performance in their study. The network uses Fully Convolutional DenseNets (FC-DenseNets) [9] for the encoder–decoder model. However, the model lacks the attention mechanism that is the key to improving accuracy. To improve the accuracy, we have embedded the channel attention into our FC-DenseNets inspired by Zhang et al.’s model [24] for spatial super-resolution.

#### 3.2.1. Multiple-Branch Network

**Figure 3:**
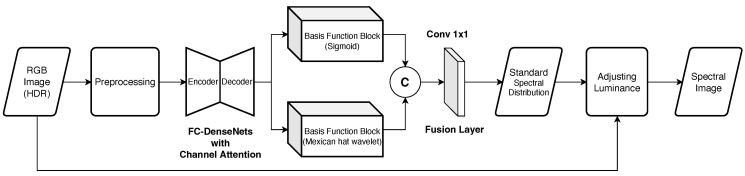
The architecture of the multiple-branch network.

We employed Mikamoto et al.’s multiple-branch network for SSR [16]. Figure 3 shows the architecture of the multiple-branch network. The overall process is as follows:

1. Extract features by the FC-DenseNets consisting of the encoder–decoder model after preprocessing HDR-RGB images;
2. Extract new features by each basis function block;
3. Generate standard spectral distribution by the fusion layer where features outputs from the two basis function blocks are concatenated.

**Figure 4:**
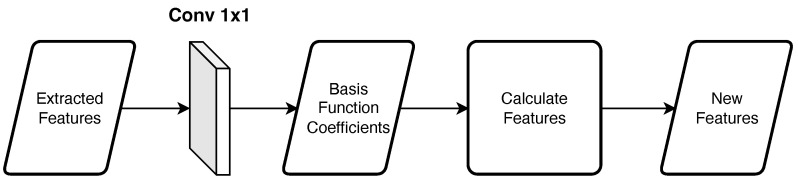
Overview of the basis function block.

Figure 4 shows an overview of the basis function block. By using the assigned basis functions, the block calculates new features. First, in the 1×1 convolution layer, the basis function coefficients are calculated for each pixel from the extracted features. Then, new features are calculated from the coefficients and basis functions. Using the following equation:

(3) $$F\left(\lambda \right)=\sum_{i=1}^{n}w_{i}f_{i}\left(\lambda \right),$$

where λ is the wavelength, *n* is the number of bases (n=10 in our experiments), $w_{i}$ is the *i*-th weights, and $f_{i}$ is the *i*-th basis. The same basis function is used in the same block. Note that the coefficients of each basis are different. We used the sigmoid function and Mexican hat wavelet as the basis function.

**Sigmoid function**

This function is used by Jakob and Hanika [25] to represent the spectrum, and the equation is as follows:

(4) $$\begin{array}{cc} f(\lambda ) & =S(c_{0}\lambda^{2}+c_{1}\lambda +c_{2}) \end{array}$$

(5) $$\begin{array}{cc} S(x) & =\frac{1}{2}+\frac{x}{2\sqrt{1+x^{2}}}, \end{array}$$

where λ is the wavelength and $c_{i}$ are the coefficients that the network learns.

**Mexican hat wavelet**

We used the following equation:

(6) $$f\left(\lambda \right)=\frac{1}{\sqrt{a}}\left(1-2{\left(\frac{\lambda -c}{a}\right)}^{2}\right)\exp\left(-{\left(\frac{\lambda -c}{a}\right)}^{2}\right),$$

where λ is the wavelength, and *a* and *c* are the coefficients that the network learns.

#### 3.2.2. Channel Attention Embedded into FC-DenseNets

**Figure 5:**
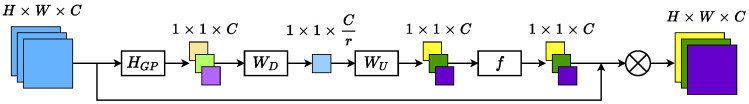
The architecture of the channel attention.

We have embedded Zhang et al.’s channel attention (see Figure 5) in our FC-DenseNets. The process of this channel attention mechanism is as follows:

1. The average value of each channel is calculated by global average pooling;
2. The number of channels is reduced to $\frac{C}{r}$ by the 1×1 convolution layer, where *r* is a hyperparameter as the reduction ratio (in this study, r=4);
3. The number of channels is restored by the 1×1 convolution layer after applying the activation function ReLU;
4. The weights among each channel are calculated using a sigmoid function, and the output values are multiplied by the input features.

**Figure 6:**
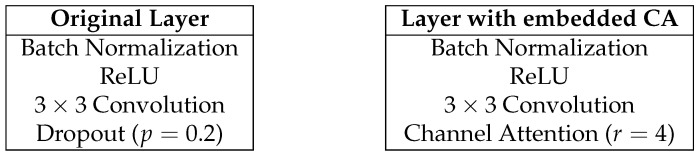
Composition of the dense layer.

Figure 6 shows the composition of the dense layer. The left part shows the original composition, while the right shows the composition in the proposed method.

### 3.3. Only Learning Spectral Similarity

When training the network, we should consider the number of samples in the dataset and the number of images with high-luminance areas such as light sources. Neural networks based on end-to-end learning require a large number of samples and images because the networks have to learn both luminance and spectral similarity simultaneously.

In the proposed method, the network learns only spectral similarity using the chrominance decomposition proposed by Sakamoto et al. [14]. SSR based on the chrominance decomposition provides good accuracy even when the number of samples and images is relatively small.

Sakamoto et al. [14] have experimentally shown that scaling the luminance using the sum of the XYZ components is the best for the luminance restoration of spectral images. Based on this result, the proposed method recovers the luminance by the following equation:

(7) $$HS_{HDR}=\frac{X_{rgb}+Y_{rgb}+Z_{rgb}}{X_{S}+Y_{S}+Z_{S}}S,$$

where $HS_{HDR}$ is the luminance-restored HDR spectral image as the final output of our method, $X_{rgb},Y_{rgb},Z_{rgb}$ are the XYZ components of an input HDR-RGB image, *S* is a standard spectral image output from the fusion layer, and $X_{S},Y_{S},Z_{S}$ are the XYZ components of the standard spectral images.

### 3.4. Creating Real HDR-HS Dataset

We trained and evaluated the network using pseudo-HDR-RGB images created from the ARAD HS dataset [12]. However, the practicality of the network trained by these images has yet to be discovered. Hence, we created a real HDR-HS dataset and evaluated the proposed method by the dataset.

#### 3.4.1. Capturing HS Images

We used a hyperspectral camera, “Specim IQ”, to take HS images. The camera can take an HS image with 512×512 spatial resolution and 204 spectral bands (400 to 1000 nm). It takes several tens of seconds to a few minutes to capture a single HS image because of the line-scan camera. For HDR synthesis, we took three HS images with different exposure times per scene. Then, we created the “Multiple Exposure HyperSpectral (ME HS)” dataset with 96 scenes.

#### 3.4.2. HDR Synthesis of HS Images

HDR-HS images were created by HDR synthesis of multiple HS images with different exposures. We employed an HDR synthesis method developed by Debevec and Malik [26]. This method estimates a non-linear camera response function and converts multiple exposure images into a single HDR image using the camera response function.

We performed HDR synthesis by manually adjusting the hyperparameters and created 82 HDR-HS images, excluding failed synthesis scenes due to moving objects such as clouds and sea surfaces. Then, an HDR-RGB image paired with the HDR-HS image was created in the same way as in Section 3.1.

**Figure 7:**
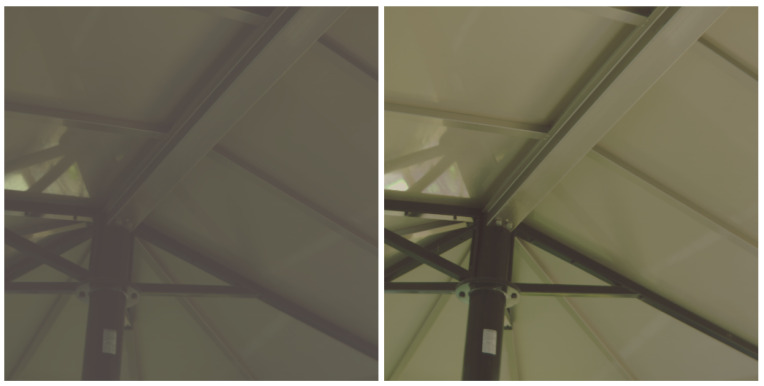
Comparison of LDR and HDR images. The left is LDR-HS, and the right is HDR-HS image.

**Figure 8:**
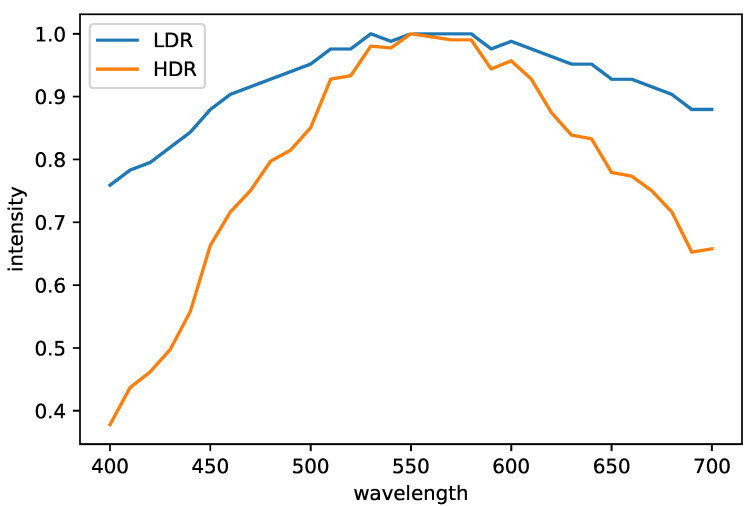
The plot of the LDR and HDR spectra. For comparison, we normalized the spectra so that the maximum value is one.

Figure 7 shows a pair of images before and after HDR synthesis, and Figure 8 plots the spectrum of a pixel in those images. The Debevec and Malik’s HDR synthesis method is also effective for HS images.

### 3.5. Extending the PBRT Renderer

We extended the PBRT renderer [2] to handle HDR-HS images generated by SSR methods as environment maps for spectral IBL. The procedure of spectral IBL is as follows:

1. Read sequentially numbered grayscale OpenEXR images for each wavelength instead of an RGB image;
2. Store them as an image array of the SampledSpectrum class, representing the PBRT renderer’s spectrum;
3. Evaluate directly as spectral values during the light source sampling process.

The sequentially numbered grayscale images consist of 31 images sampled at 10 nm intervals over the wavelength range of 400 to 700 nm. Before the extension, the PBRT renderer stores spectrum converted the inputting RGB image by the method of Smits [20] in SampledSpectrum class. On the other hand, the extended PBRT renderer directly stores HS images in the class and only performs spectral rendering.

### 3.6. Experiments

#### 3.6.1. Dataset

We trained and evaluated the network using the ARAD HS dataset [12] and further evaluated its performance using the ME HS dataset created in Section 3.4.

The ARAD HS dataset was used for the “Spectral Reconstruction from an RGB Image—Track 1 Clean” in the “New Trends in Image Restoration and Enhancement workshop and challenges on image and video restoration and enhancement 2020”. It consists of 510 data in total:

- 450 training data;
- 30 validation data (only 10 are open to the public);
- 30 test data (test data are not open to the public).

The image size and the number of spectral bands are 512×482 and 31 (400 to 700 nm in 10 nm steps), respectively. Since the test data are not publicly available, we used the official validation data as test data and randomly selected 10 data from the training data as validation data in our experiment.

The ME HS dataset consists of 96 scenes which have three HS images with different exposures per scene. For evaluating a network, each scene was performed HDR synthesis. Created HDR-HS images have 512×512 spatial resolution and 31 spectral bands (400 to 700 nm in 10 nm steps). We used 82 HDR-HS images to evaluate a network.

#### 3.6.2. Implementation Details

We trained the networks for 100 epochs using Adam optimizer [27] and used cosine annealing [28] as learning rate decay (parameters: $\eta_{max}=0.002$, $\eta_{min}=0.0001$, $T_{0}=20$, $T_{mult}=1$). For data augmentation, we used random horizontal flip (p=0.5), random vertical flip (p=0.5), and random rotation ($90^{\circ },270^{\circ },p=\left[\frac{1}{3},\frac{1}{3}\right]$).

The proposed method uses Reinhard’s tone mapping as preprocessing to normalize the luminance of input HDR-RGB images. In order to verify the effectiveness of the preprocessing, we experimented with two different cases: with and without preprocessing. In the case of “with preprocessing”, HDR-RGB images are processed using Reinhard’s tone mapping, and luminance-normalized images are input to the network. In the case of “without preprocessing”, HDR-RGB images are directly input to the network.

For comparison, Galliani et al.’s method [5], Shi et al.’s method (HSCNN-D) [11], and Mikamoto et al.’s two-branch network with sigmoid function and Mexican hat wavelet [16] were also trained in the same way. In addition, Li et al.’s method (AWAN) [13] was trained in the same way except for the cosine annealing (modified parameters: $\eta_{max}=2\times 10^{-4},\eta_{min}=1\times 10^{-5},T_{0}=20,T_{mult}=1$) because the cosine annealing did not work well.

Furthermore, we compared our method with Smits’s method [20], an SSR method that does not use neural networks and is used in the PBRT renderer [2].

#### 3.6.3. Evaluation Metrics

We used a Spectral Angle Mapper (SAM) to evaluate the similarity of spectral distributions and Mean Relative Absolute Error (MRAE) to evaluate the error of spectral intensity values. These evaluation metrics are calculated as follows:

(8) $$\begin{array}{cc} SAM & =\frac{1}{N}\sum_{i=1}^{N}\arccos\frac{{\hat{y}}_{i}\cdot y_{i}}{∥{\hat{y}}_{i}{∥}_{2}{∥y_{i}∥}_{2}}, \end{array}$$

(9) $$\begin{array}{cc} MRAE & =\frac{1}{N}\sum_{i=1}^{N}\sum_{c=1}^{C}\frac{|{\hat{y}}_{i,c}-y_{i,c}|}{y_{i,c}}, \end{array}$$

where $y_{i}$ and $\hat{y_{i}}$ are the ground truth and the estimated spectrum of the *i*-th pixel, respectively; *N* is the total number of pixels, and *C* is the total number of channels in the spectrum. The unit of SAM after the calculation is radians, and we converted from radians to degrees in the evaluation. For both of the evaluation metrics, smaller values indicate better performance.

## 4. Results

### 4.1. Comparison of Learning Methods

We investigated the effectiveness of the proposed method for learning spectral similarity. In end-to-end learning, we changed the loss function to mean squared error. Table 1 shows the quantitative results of the proposed and Galliani’s network trained by two different learning methods. Both methods of learning spectral similarity outperformed the end-to-end learning in both datasets. In addition, the MRAE of the end-to-end learning on the ME HS dataset was much worse than the proposed method because the training dataset does not contain the luminance distributions of the ME HS dataset. This result indicates that the proposed method of learning spectral similarity can flexibly deal with the luminance distributions different from the training dataset and is extremely useful for HDR spectral super-resolution.

### 4.2. Quantitative Evaluation

Table 2 shows the quantitative results obtained for each method on the ARAD HS dataset. The proposed multiple-branch network with embedded channel attention (ours) outperformed the conventional methods. Table 2 also shows that the preprocessing with Reinhard’s tone mapping provides better results than directly inputting HDR into the network.

In addition, we evaluated on the real HDR-HS images for practical applications. Table 3 shows the results on the ME HS dataset. The proposed method had top-class performance in practical usage. However, the preprocessing only worked well with the proposed method. It can also be seen that large models such as the AWAN and HSCNN-D were less accurate than the methods of Mikamoto et al. and Galliani et al., which have smaller models. The large models seem more adapted to the ARAD HS dataset and caused deterioration of generalization performance. It can be said that the small model is more practical than the large model in the current situation with fewer training data.

We also examined how effectively the proposed method works for LDR-RGB images. Table 4 shows the results when LDR-RGB images are input to the networks trained with HDR images. The images used for evaluation were converted from the spectral images in Table 2. The proposed method had the best result in MRAE, although it had the second-best result in SAM. The result also shows that it is possible to deal with LDR images in the same way as HDR images by introducing preprocessing to normalize the luminance of the input images. This means the same network can be used for input HDR and LDR images.

Table 5 shows the conversion time from RGB to HS for each method. The conversion time is the average of ten images with a size of 2048×1024. The method of Smits, the SSR method that does not use deep learning, was the fastest. In the case of CPU, the conversion time of SSR methods with large network models such as AWAN and HSCNN-D was quite long. On the other hand, the conversion time can be accelerated by using GPU. When using GPU, the proposed method had a top-level conversion speed comparable to the speed of the non-network-based method.

### 4.3. Qualitative Evaluation

**Figure 9:**
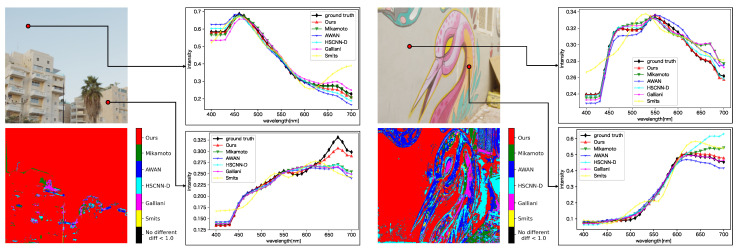
Qualitative evaluation of each method on the ARAD HS dataset. For each sub-figure, the upper left is the input image, the lower left shows the most accurate method for each pixel based on the evaluation metric SAM, and the upper right and lower right plot the spectral distributions for the selected pixels.

Figure 9 shows a comparison of the spectral distributions generated by each method. For each sub-figure, the upper left is the input image, the lower left shows the most accurate method for each pixel based on the evaluation metric SAM, and the upper and lower right plot the spectral distributions for the selected pixels. The lower left sub-figures show that the proposed method generated better results than the other methods in many regions. From the plot of spectral distributions, it can be seen that the proposed method reconstructed spectral distributions better than the other methods, especially in the long wavelength regions. In addition, the proposed method produced more stable spectral distributions because it produced relatively good spectra even in regions where the other methods gave better results than the proposed method.

**Figure 10:**
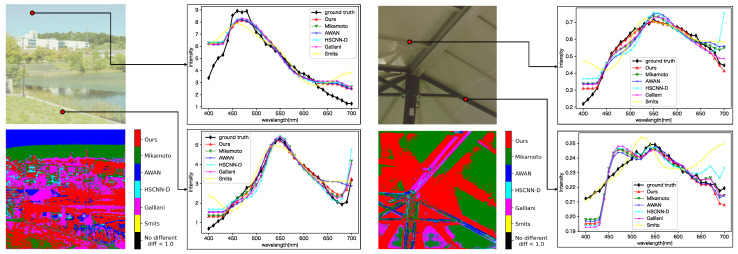
Qualitative evaluation of each method on the ME HS dataset. For each sub-figure, the upper left is the input image, the lower left shows the most accurate method for each pixel based on the evaluation metric SAM, and the upper right and lower right plot the spectral distributions for the selected pixels. The evaluation of each method is based on the best result among the cases with and without preprocessing.

We also evaluated each method on the ME HS dataset (see Figure 10). The proposed method generated better results on the real-HDR HS images, but the difference was smaller than on the ARAD HS dataset. All SSR methods generated constant spectral distributions in the 400 to 440 nm wavelength. The reason is that the ARAD HS dataset used for training the network tends to take constant distributions in the wavelength region.

### 4.4. Spectral Rendering

We performed spectral IBL using the extended PBRT renderer [2] as a practical example.

**Figure 11:**
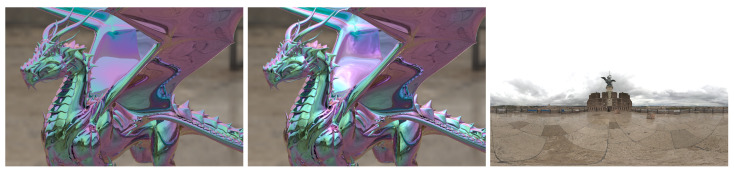
Comparison of IBL rendering with LDR and HDR environment maps. The left image is rendered with an LDR-HS environment map, the middle image is rendered with an HDR-HS environment map, and the right image is the environment map used for spectral IBL rendering.

First, we compared the rendering results using the proposed HDR SSR method with those using the conventional LDR SSR method. Figure 11 shows the result of the spectral IBL rendering with a conventional LDR SSR and the proposed HDR SSR methods. The HS image for the environment map consists of 30 segments of the 400 to 700 nm wavelength range at 10 nm intervals, and in spectral rendering, path tracing with sampling 1024 rays per pixel was used. The object’s material is silicon coated with multilayer thin films (a 400 nm thick ${\mathrm{SiO}}_{2}$ film and a 5-nm-thick aluminum film on the surface). The dragon model used in rendering is available in [29,30]. Although IBL with an environment map generated by the LDR SSR method can render the effects of thin-film interference, the image quality of the highlight areas was degraded by the limited dynamic range. By contrast, the HDR-HS environment map generated by the proposed method yielded a higher-quality image.

**Figure 12:**
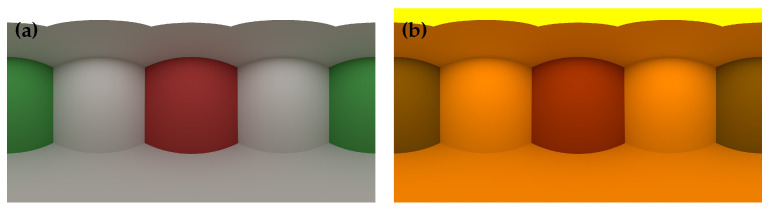
Environment maps for spectral IBL. (**a**) D65. (**b**) HP1.

Next, we compared the original PBRT renderer with the extended PBRT renderer, which combined with our SSR method. The original PBRT renderer performs spectral rendering using a Smits’s method [20], where an HDR-RGB input is upsampled to the spectrum. Figure 12 shows the environment maps with different light sources. We created the environment maps by spectral rendering the Cornell box with the PBRT render. As the light source, we set a D65 and a standard high pressure sodium lamp (HP1).

**Figure 13:**
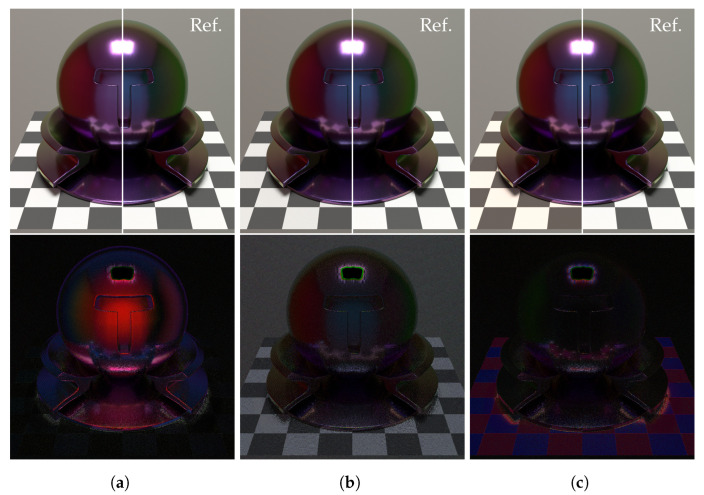
Results of rendering (D65). (**a**) RGB rendering. (**b**) PBRT renderer. (**c**) Ours.

**Figure 14:**
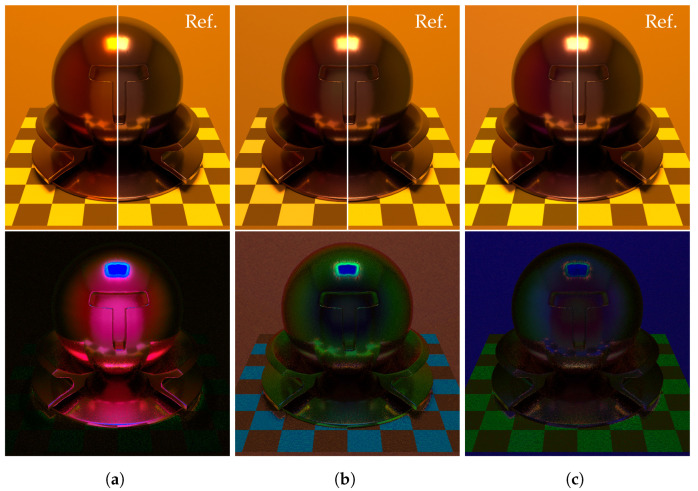
Results of rendering (HP1). (**a**) RGB rendering. (**b**) PBRT renderer. (**c**) Ours.

Figure 13 and Figure 14 show the rendered results for each method. Spectral environment map consists of 30 segments of the 400 to 700 nm wavelength range at 10 nm intervals, and path tracing was used in spectral rendering with sampling 128 rays per pixel. The object’s material is silicon coated with a 300 nm thick ${\mathrm{SiO}}_{2}$ film. In each figure, the top row shows the rendered results for each method, and half of the right is the reference image that is the result of the spectral rendering with full 3D objects. The bottom row shows the difference images between the rendering results and the reference image. The intensity of the different images is multiplied by 5 for easy observation.

From these figures, the RGB rendering cannot render the accurate colors of the surroundings reflected on the object. Comparing the proposed method to the PBRT renderer, the proposed method can generate better results in colors of the surroundings reflected on the object because the proposed method renders interference effects with an accurate spectral environment map. However, the proposed method has larger numerical errors than RGB rendering in the region of the checkerboard. The reason is that the proposed method cannot represent a light source spectrum well because the training dataset has a few samples of light sources.

## 5. Discussion

Conventional SSR methods generate an HS image from an RGB image and make HS images more accessible. However, some practical applications require HDR images, while conventional SSR methods target LDR images.

We have developed an SSR method that supports HDR images. The proposed method reconstructs more accurate spectra in HDR than conventional SSR methods. The preprocessing, which normalizes a luminance, allows for handling both LDR and HDR images and is effective in the proposed HDR SSR method.

### Limitations

The proposed method is capable of handling any HDR-RGB images. However, it should be noted that the dynamic range of the output HS images is limited by that of the training HS images used in the model. Therefore, if we want to generate HS images with a wider dynamic range, we need to prepare a dataset that covers a wider dynamic range.

## 6. Conclusions

In this paper, we proposed an SSR method using deep learning for HDR images. The proposed method with the preprocessing reconstructs HDR-HS images from HDR-RGB images more accurately and is compatible with LDR-RGB images. Additionally, we demonstrated a practical use of the proposed method combined with a spectral IBL. The rendering results showed that the proposed HDR SSR method combined with spectral rendering enables to render of realistic images with highly wavelength-dependent optical phenomena.

As a future work, we are planning to accelerate the SSR method to improve its performance. Enhancing the spectral resolution and conversion of HDR-HS images from LDR-RGB images are also our future work. Further improvement of conversion accuracy in the region of short wavelength and light source spectra are challenging problems to be solved.

## Figures and Tables

**Table 1:** Comparison of learning methods. ⋄ indicates learning spectral similarity, and e2e indicates end-to-end learning.

	ARAD HS		ME HS	
Method	SAM ↓	MRAE ↓	SAM ↓	MRAE ↓
Ours ⋄	2.155	0.0342	5.509	0.1066
Ours (e2e)	3.406	0.0559	7.305	0.9028
Galliani ⋄	2.784	0.0423	6.089	0.1135
Galliani (e2e)	4.248	0.0774	7.955	0.8985

**Table 2:** Quantitative evaluation of the experiments on the ARAD HS dataset. Bolded and underlined indicate the best and second-best results, respectively.

	With Preprocessing		Without Preprocessing	
Method	SAM ↓	MRAE ↓	SAM ↓	MRAE ↓
Ours	**2.155**	**0.0342**	2.914	0.0441
Mikamoto	2.894	0.0445	2.955	0.0436
AWAN	2.790	0.0418	2.812	0.0411
HSCNN-D	2.753	0.0403	2.778	0.0398
Galliani	2.784	0.0423	2.760	0.0413
Smits	-	-	5.932	0.0934

**Table 3:** Results on the ME HS dataset. Bolded and underlined indicate the best and second-best results, respectively.

	With Preprocessing		Without Preprocessing	
Method	SAM ↓	MRAE ↓	SAM ↓	MRAE ↓
Ours	**5.509**	0.1066	6.460	0.1152
Mikamoto	5.995	0.1105	5.611	**0.1059**
AWAN	6.529	0.1281	6.404	0.1274
HSCNN-D	6.460	0.1214	6.415	0.1177
Galliani	6.089	0.1135	5.723	0.1067
Smits	-	-	6.392	0.1319

**Table 4:** The results when LDR-RGB images are input to the networks trained with HDR images. Bolded and underlined indicate the best and second-best results, respectively.

	With Preprocessing		Without Preprocessing	
Method	SAM ↓	MRAE ↓	SAM ↓	MRAE ↓
Ours	2.518	**0.0429**	3.560	0.0587
Mikamoto	3.281	0.0561	3.650	0.0692
AWAN	**2.498**	0.0439	2.592	0.0443
HSCNN-D	3.746	0.0574	3.731	0.0586
Galliani	3.654	0.0639	4.027	0.0685
Smits	-	-	9.245	0.1436

**Table 5:** Comparison of conversion time from RGB to HS (the unit is seconds). CPU is Intel Core i9-11900, and GPU is NVIDIA GeForce RTX 3090.

Method	CPU	GPU
Ours	16.338	1.066
Mikamoto	14.766	0.948
Galliani	6.337	0.946
HSCNN-D	51.998	2.344
AWAN	324.586	7.110
Smits	0.788	-

## Data Availability

The data presented in this study are available on request from the corresponding author.

## Abbreviations

The following abbreviations are used in this manuscript:

HS	Hyperspectral
LDR	Low Dynamic Range
HDR	High Dynamic Range
IBL	Image-Based Lighting
CNN	Convolutional Neural Network
SSR	Spectral Super-Resolution
